# A Comparative Study of Breast Tumors Before and After Neoadjuvant Therapy

**DOI:** 10.3390/diagnostics16213548

**Published:** 2026-10-28

**Authors:** Adeline-Roxana Bucur, Alexandru Cristian Cindrea, Antonia Armega-Anghelescu, Paul Szeica, Crina-Andreea Porumbel, Alina Cristina Barb, Florin Emil Hut, Flavia Zara

**Affiliations:** 1 Doctoral School, “Victor Babes” University of Medicine and Pharmacy Timisoara, E. Murgu Square, No. 2, 300041 Timisoara, Romania; adeline.bucur@umft.ro (A.-R.B.); antonia.armega@umft.ro (A.A.-A.); paul.szeica@umft.ro (P.S.); 2 Department of Pathology, Emergency City Hospital, 300254 Timisoara, Romania; crina.andreea@gmail.com (C.-A.P.); flavia.zara@umft.ro (F.Z.); 3 Centre for Artificial Intelligence and Big Data in Emergency Medicine and Clinical Research, Department of Surgery, “Victor Babes” University of Medicine and Pharmacy Timisoara, 300041 Timisoara, Romania; 4 Department of Microscopic Morphology, “Victor Babes” University of Medicine and Pharmacy Timisoara, 300041 Timisoara, Romania; toma.alina@umft.ro; 5 University Clinic of Surgery I, “Victor Babes” University of Medicine and Pharmacy Timisoara, 300041 Timisoara, Romania; florin.hut@umft.ro; 6 Center for Hepato-Bilio-Pancreatic Surgery, “Victor Babes” University of Medicine and Pharmacy Timisoara, 300041 Timisoara, Romania; 7 Angiogenesis Research Center, “Victor Babes” University of Medicine and Pharmacy Timisoara, E. Murgu Square, No. 2, 300041 Timisoara, Romania

**Keywords:** breast cancer, neoadjuvant therapy, pathological response, molecular subtype, residual cancer burden, tumor-infiltrating lymphocytes

## Abstract

**Background:** Pathological response after neoadjuvant therapy in breast cancer varies substantially across immunohistochemical surrogate subtypes. This study aimed to compare pretreatment biopsy features with post-treatment surgical pathology findings and to explore baseline predictors of favorable pathological response. **Methods:** We retrospectively analyzed female patients with invasive breast carcinoma treated with neoadjuvant therapy followed by surgery at a single institution between January 2020 and December 2024. Pretreatment histological features, stromal tumor-infiltrating lymphocytes, tumor cellularity, necrosis, in situ component, lymphovascular/perineural invasion, and immunohistochemical surrogate subtype were compared with post-treatment findings. Residual disease was assessed using the Residual Cancer Burden system, and vital status was analyzed as an exploratory outcome. **Results:** The analytical dataset included 146 breast carcinoma records. RCB-0, RCB-I, RCB-II, and RCB-III were observed in 20, 14, 64, and 46 of 144 evaluable records, respectively, and pCR was identified in 18/142 evaluable records. Tumor cellularity decreased significantly after therapy, whereas stromal TILs and tumor necrosis did not change significantly. Favorable pathological response and pCR were more frequent in HER2-positive and triple-negative tumors than in luminal tumors. In univariable Firth regression, higher baseline stromal TILs and HER2-positive/triple-negative subtype were associated with increased odds of RCB 0/I and pCR, and both associations persisted in multivariable models adjusted for age and grade. Nineteen patients (13.0%) deceased; crude mortality increased across RCB classes (0%, 14.3%, 7.8% and 26.1% for RCB-0 to RCB-III; *p* = 0.008), with no deaths among patients achieving pCR. **Conclusions:** In conclusion, favorable pathological response after neoadjuvant therapy was uncommon in this cohort and was observed mainly in HER2-positive and triple-negative tumors. Baseline stromal TILs and immunohistochemical surrogate subtype showed exploratory associations with response, supporting their further evaluation in larger, treatment-stratified cohorts with formal survival follow-up.

## 1. Introduction

Breast cancer (BC) is the most commonly diagnosed malignancy among women worldwide and remains a leading cause of cancer-related mortality [1]. Breast cancer is commonly associated with environmental, reproductive, and lifestyle factors, some of which are potentially modifiable [2].

Survival depends on both stage and molecular subtype [3]. Estrogen receptor (ER) status, progesterone receptor (PR) status, human epidermal growth factor receptor-2 (HER2) status, Ki-67 proliferation indices, and multigene panels all play a contributive role in the personalization of treatment modalities for each case [4]. Invasive BC can be divided into four principal surrogate molecular subtypes by immunohistochemistry based on the expression of ER, PR, HER2 and the Ki-67 proliferation index [5]. Luminal A BC (ER-positive and PR-positive, HER2-negative, with a low Ki-67 index) represents around 60% of BC and is associated with a good prognosis [6]. Luminal B BC (ER-positive, with low or absent PR expression and/or a high Ki-67 index, either HER2-negative or HER2-positive) represents about 30% of BC and is associated with a poorer prognosis than luminal A tumors [7]. HER2-positive (non-luminal) tumors lack hormone receptor expression and overexpress HER2, whereas triple-negative tumors express none of the three receptors [5].

Systemic therapy is subtype-driven and may include chemotherapy, endocrine therapy, HER2-targeted therapy, immunotherapy, or combinations thereof, depending on stage, biological subtype and drug availability [8]. Although the majority of patients present with localized disease, about 25–40% eventually develop metastatic disease which, in spite of advances in treatment strategies, is still largely incurable [9].

For patients diagnosed with breast cancer, whole breast irradiation (WBI) following local excision of the primary tumor (lumpectomy) is a fundamental part of contemporary multimodality management of breast cancer. This approach, known as breast-conserving therapy (BCT) has shown optimal loco-regional control and equivalent overall and disease-free survival compared to mastectomy alone in early breast cancer [10]. Use of modern neoadjuvant therapy (NAT) regimens has markedly increased rates of pathologic complete response (pCR) in breast cancer [11]. Targeted anti-HER-2 therapy (transtuzumab) and tyrosine kinase receptor inhibitors (TKIs) (lapatinib) have achieved treatment effects in HER2-positive breast tumors [12]. The advent of immunotherapy and antibody–drug conjugates (ADCs) has changed the landscape of BC, especially triple-negative BC (TNBC), for which conventional treatment options are limited [13]. Immunotherapy, in the form of PD-1 and PD-L1 inhibitors, is now a therapeutic option in BC that can improve survival amongst responders, especially in local advanced or metastatic TNBC [14,15]. Even if tumors that show greater immunogenicity and have greater infiltration of immune cells tend to be an indicator of response to chemotherapy and good prognosis, especially in TNBC and HER2-amplified BC [16], breast cancer subtypes have not shown significant response to current immunomodulatory therapies [17]. Breast cancer of certain subtypes may have an excellent chemosensitivity but may also show a spectrum of post-NAT residual disease ranging from minimal (near pathologic complete response) to extensive residual disease [18].

The index score is derived from the largest area and cellularity of residual invasive primary cancer and the number of involved lymph nodes and size of largest metastasis. pCR (stage yp-T0/is, ypN0) has a residual cancer burden (RCB) of 0. The RCB is classified as minimal (RCB-I), moderate (RCB-II), or extensive (RCB-III) [19].

This study aimed to compare pre- and post-neoadjuvant therapy pathological findings in patients with invasive breast carcinoma treated at a single institution. Specifically, we quantified residual disease using the RCB system and explored whether baseline histological type, Nottingham grade, stromal tumor-infiltrating lymphocytes, tumor cellularity, necrosis, in situ component, lymphovascular/perineural invasion, and immunohistochemistry-based surrogate subtype were associated with pathological complete response and favorable pathological response, defined as RCB 0/I. We further assessed therapy-associated changes in selected histopathological and biomarker variables in paired pre- and post-treatment specimens. We hypothesized that higher baseline stromal TILs and HER2-positive or triple-negative surrogate subtype would be associated with improved pathological response. Finally, we explored whether pathological response and baseline features were associated with vital status.

## 2. Materials and Methods

### 2.1. Study Design

**Figure 1:**
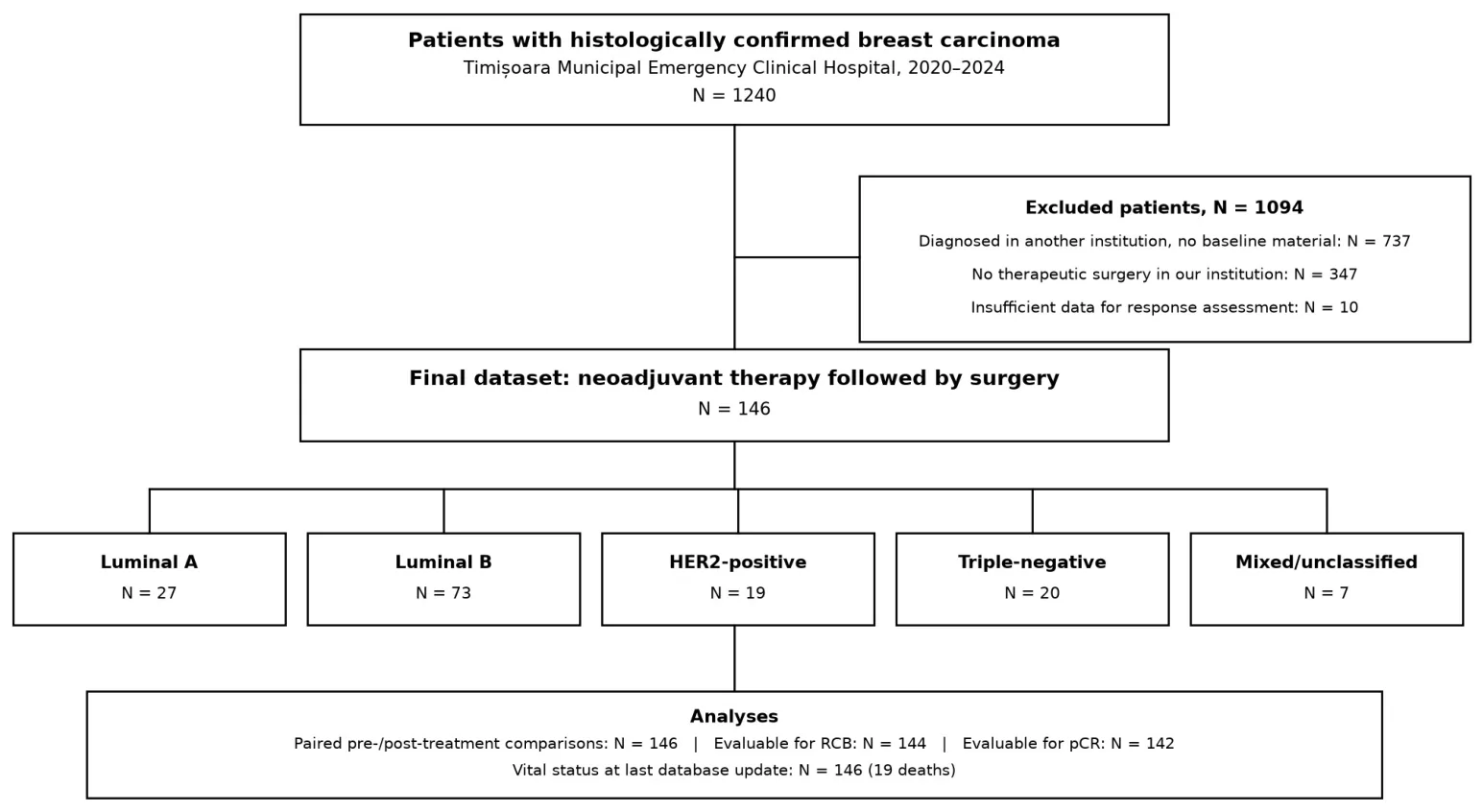
Study flowchart.

This retrospective and comparative single-center study included all the patients who were admitted to either the Surgical Oncology or the General Surgery Departments of the Timișoara Municipal Hospital between 1 January 2020 and 31 December 2024. Patient data were obtained from the electronic medical records. The study flowchart is presented in Figure 1.

The study cohort encompassed all identified patients with invasive breast carcinoma, with clinical characteristics—including age at diagnosis, intervention modalities, neoadjuvant therapy administration, and post-treatment pathological tumor staging—carefully documented.

Initial diagnosis was predominantly established via core needle (trucut) or incisional biopsy. For the core-needle and incisional biopsies, a representative slide was chosen and the following features were assessed in standard H&E stain: type of tumor, differentiation, percentage of tumor cells in the tumor bed, percentage of tumor infiltrating lymphocytes in the stroma, the presence of necrosis, in situ carcinoma, angioinvasion or perineural invasion. Differentiation was assessed based on the Nottingham score, a 3-tier system taking into consideration gland formation, nuclear pleomorphism, and mitotic activity [20].

TILs were analyzed both as a continuous parameter and in three predefined groups: low (sTILs 0–10%), intermediate (sTILs 11–59%), and high (sTILs ≥ 60%) [21].

Further assessment of hormonal receptors, HER2 status and Ki-67 index was made on the pretreatment biopsy, with assignment to immunohistochemistry-based surrogate subtypes according to the St Gallen surrogate definitions [22], using the following algorithm: Luminal A, ER-positive and PR-positive, HER2-negative, Ki-67 ≤ 14%; Luminal B, ER-positive and either HER2-positive (irrespective of PR and Ki-67) or HER2-negative with Ki-67 > 14% and/or PR-negative; HER2-positive (non-luminal), ER-negative and PR-negative with HER2 overexpression/amplification; and triple-negative, ER-, PR- and HER2-negative. Thus, the ‘HER2-positive’ group comprised exclusively hormone receptor-negative tumors, whereas hormone receptor-positive/HER2-positive tumors were classified as Luminal B. ER, PR, HER2, and Ki-67 were interpreted according to current ASCO/CAP recommendations and institutional protocols. ER and PR positivity were defined as nuclear staining in ≥1% of invasive tumor cells. HER2 IHC was scored as 0, 1+, 2+, or 3+; cases scored 2+ were considered equivocal and were reflex-tested by in situ hybridization. Ki-67 was assessed in invasive tumor cells, preferentially in hot-spot areas, and reported as a percentage. Tumors whose immunohistochemical profile could not be assigned to a single category because of heterogeneous or discordant receptor expression between tumor components were recorded as ‘mixed/ambiguous’ (n = 2), and tumors for which IHC data were not available in the pathology archive were recorded as ‘unclassified’ (n = 5). Both groups were retained in the cohort because the study was designed to include all consecutive eligible patients and to describe the cohort transparently, but they were excluded from the subtype-specific comparisons and from the regression models.

Neoadjuvant treatment in our institution followed the NCCN guideline recommendations in use at the time of diagnosis [23]. Regimens were selected by the treating oncologists according to biological subtype and clinical stage. In general, HER2-positive tumors received taxane- and platinum-based chemotherapy combined with HER2-targeted therapy (trastuzumab, with pertuzumab in selected cases; TCHP regimen); triple-negative tumors received sequential anthracycline- and taxane-based chemotherapy, with pembrolizumab added in eligible patients once it became available; most Luminal B tumors received sequential anthracycline- and taxane-based chemotherapy; and selected hormone receptor-positive, HER2-negative tumors (all postmenopausal Luminal A patients and three premenopausal patients) received neoadjuvant endocrine therapy with an aromatase inhibitor (letrozole) or, in selected cases, tamoxifen.

According to the medical records, neoadjuvant treatment was documented as incomplete in only two patients. The recorded regimens were standard, including, for HER2-positive tumors, six cycles of docetaxel, carboplatin, trastuzumab and pertuzumab (TCHP) every 3 weeks, or four cycles of doxorubicin/epirubicin plus cyclophosphamide (AC/EC) followed by 12 weekly doses of paclitaxel with trastuzumab, with or without pertuzumab; for triple-negative tumors, four cycles of dose-dense AC/EC followed by 12 weekly doses of paclitaxel, with the addition of carboplatin and pembrolizumab after its approval (KEYNOTE-522 regimen); and for hormone receptor-positive/HER2-negative tumors, four cycles of AC/EC followed by 12 weekly doses of paclitaxel or four cycles of docetaxel, or neoadjuvant endocrine therapy with an aromatase inhibitor for at least 4–6 months in selected patients. Because the study database was built from the pathology archive, individual-level treatment data (specific agents, number of cycles, dose modifications and completion of treatment) and pretreatment clinical (cTNM) stage could not be retrieved for each patient; treatment is therefore described at the level of the subtype-specific protocol and was not analyzed as a patient-level variable.

The resection specimens were further evaluated, and the staging of the tumors was done according to the latest AJCC using the TNM (Tumor, Node, Metastasis) system, with further subdivision into pathological stages. In evaluating the response to therapy, the AJCC/TNM system was adopted, taking into consideration the largest contiguous focus of the invasive cancer in determining ypT stage in ypTNM system [24].

The carcinomas were divided in the following pathological stages: stage 0 (DCIS or ypT0 tumors), stage IA (pT1N0), stage IB (pT0–1N1mi), stage IIA (pT0–1N1, pT2N0), stage IIB (pT2N1, pT3N0), stage IIIA (pT0–2N2, pT3N1–2), stage IIIB (pT4N0–2), stage IIIC (anyTN3), stage IV (any T, any N, M1) [25].

RCB was calculated using the MD Anderson Residual Cancer Burden calculator. For each post-treatment resection specimen, the largest two dimensions of the residual tumor bed were recorded macroscopically and/or microscopically. The largest cross-sectional area of the tumor bed was submitted or reviewed whenever available. Overall cancer cellularity and the percentage of in situ carcinoma were estimated across the tumor bed. The number of positive lymph nodes and the diameter of the largest nodal metastasis were recorded. RCB class was assigned as RCB-0, RCB-I, RCB-II, or RCB-III [26].

A significant number of tumors were further reassessed for molecular subtype.

### 2.2. Data Collection

Patient data were collected from the hospital electronical records.

Data regarding patients’ demographics (sex, age), surgical procedures, tumor staging, histological grading, and molecular subtype were collected using Microsoft Excel (Microsoft Office 16, Redmond, WA, USA). Immunohistochemistry (IHC) results used to determine molecular subtype were available for 141 of the 146 carcinoma cases. For the remaining five cases, molecular subtype was recorded as unclassified because of unavailable IHC data. Vital status (alive or deceased) at the time of the last database update was retrieved from the hospital records; the date of death was available for 17 of the 19 deceased patients, whereas the dates of surgery and of the last follow-up contact were not uniformly available in the pathology-based database. Data on recurrence were not available.

### 2.3. Inclusion and Exclusion Criteria

Inclusion criteria were histologically confirmed breast carcinomas and availability of sufficient clinicopathological information for analysis; for resection specimens, only cases with pathological staging were included. Patients were excluded if they were diagnosed outside our institution without available baseline pathological material, did not undergo therapeutic surgery at our institution, or had insufficient pathological data for response assessment.

### 2.4. Laboratory Technique

All the small biopsies and pathological resection specimens were sent to our department on the same day as the intervention. They were received in formalin; the resection specimens were sectioned into 0.5–1 cm thick slices for better fixation and left for a maximum of 48 h in formalin. After gross description and sampling, the tissue sections were embedded in paraffin, and four micrometer-thick sections were cut using a semi-automated *Leica RM2235* rotary microtome (Leica Biosystems, Nussloch, Germany) and displayed on *Super Frost™* microscope slides (St. Louis, MO, USA). For morphological diagnosis, the hematoxylin–eosin technique was primarily used.

The microscopic aspects were recorded for each tumor as follows: architectural pattern of growth, cell type, rate of mitotic figures, necrosis, inflammatory response, in situ component, angiolymphatic invasion, and perineural invasion.

For resection specimens, margin status was additionally assessed, and in axillary dissection specimens, each lymph node was examined separately.

Immunohistochemical staining for ER, PR, HER2, and Ki-67 was performed on the Leica Bond-Max, Leica Biosystems, Newcastle, UK automated platform using the standardized laboratory protocols recommended by the manufacturer in use during the study period. All the antibodies and reagents for immunohistochemical staining were purchased from Leica Biosystems, Newcastle, UK. ER and PR positivity were defined as staining in ≥1% of tumor cell nuclei. HER2 was interpreted using the conventional 0/1+/2+/3+ scoring system, with 0 and 1+ considered negative, 3+ considered positive, and 2+ considered equivocal. Ki-67 was assessed as the percentage of positively stained invasive tumor cell nuclei.

### 2.5. Statistical Workflow

Statistical analysis was performed in Python 3.11 (pandas, SciPy). The database was first checked for consistency, missing values, duplicate entries, and variable coding errors. Patient identifiers were anonymized, and categorical variables were recoded into standardized formats before analysis. Descriptive statistics were used to summarize the cohort. Continuous variables were reported as median and interquartile range or mean ± standard deviation, as appropriate, while categorical variables were presented as absolute frequencies and percentages. Percentages were calculated using the number of available non-missing observations for each variable as the denominator.

Paired comparisons between pre- and post-neoadjuvant therapy variables were performed using the Wilcoxon signed-rank test for continuous or ordinal variables and McNemar’s test for paired categorical variables; McNemar’s test was computed from the 2 × 2 table of paired observations using the chi-square statistic with continuity correction, and the exact binomial version is reported when the number of discordant pairs was small. The concordance between RCB-0 and pCR was verified case by case and is reported as a cross-tabulation. Associations between baseline characteristics and pathological response groups were assessed using the Mann–Whitney U test for continuous variables and Fisher’s exact test for categorical variables. Because of the limited number of favorable response events, predictors of good pathological response and pathological complete response were further explored using univariable Firth penalized logistic regression models, with results reported as odds ratios and 95% confidence intervals. Multivariable Firth models were additionally fitted for RCB 0/I and pCR. Covariates were prespecified as age (per 10 years), Nottingham grade (G3 versus G1–2), baseline sTILs (per 5-percentage-point increase) and surrogate subtype (HER2-positive/triple-negative versus luminal); the number of covariates was limited according to the number of events (grade was omitted from the pCR model), and a sensitivity model for RCB 0/I additionally included the baseline in situ component and lymphovascular invasion. Pretreatment clinical stage and treatment regimen could not be included because these variables were not available; post-treatment pathological (ypTNM) stage was not entered as a covariate because it is itself a measure of response and is collinear with the RCB outcome. Given the limited number of events, all regression analyses were considered exploratory.

Vital status was analyzed as a binary outcome. Associations between vital status and RCB class, pathological response, pCR, baseline sTILs, surrogate subtype and other baseline features were therefore explored using Fisher’s exact test (with a Monte Carlo approximation for tables larger than 2 × 2), the Cochran–Armitage test for trend across RCB classes, the Mann–Whitney U test and univariable and multivariable Firth logistic regression.

Good pathological response was defined as Residual Cancer Burden class 0/I, while poor response was defined as RCB II/III. Pathological complete response was defined as ypT0/is ypN0.

All statistical tests were two-tailed, and a *p*-value < 0.05 was considered statistically significant.

### 2.6. Ethics

The study was conducted in accordance with Romanian legislation and the ethical principles set out in the Declaration of Helsinki. Upon admission, patients routinely sign a general informed consent permitting the use of anonymized medical data for academic purposes. Nevertheless, because of the retrospective nature of the study and the exclusive use of existing clinical and pathological data, the requirement for additional informed consent was waived. Histological processing was performed in accordance with the recommendations of the Romanian Ministry of Health and applicable international protocols. Ethical approval for the study was granted by the Ethics Committee of Timișoara Municipal Emergency Clinical Hospital (Institutional Review Board Statement No. E2455/20.05.2026).

## 3. Results

**Figure 2:**
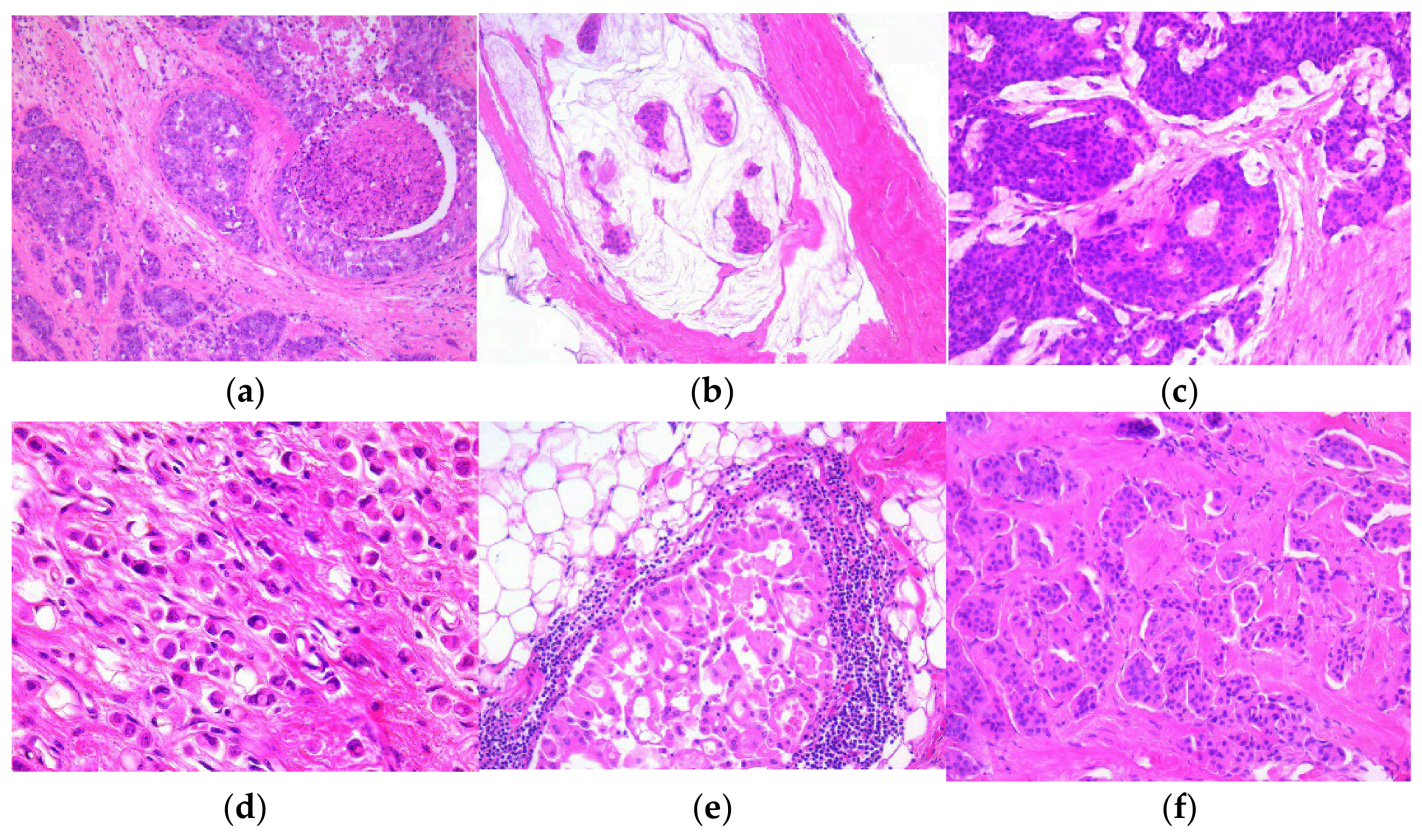
Different subtypes of breast cancer on trucut biopsy, ob 20×, H&E: (**a**) mammary invasive NST, (**b**) mucinous type A, (**c**) mucinous type B, (**d**) lobular, (**e**) apocrine, (**f**) micropapillary.

**Figure 3:**
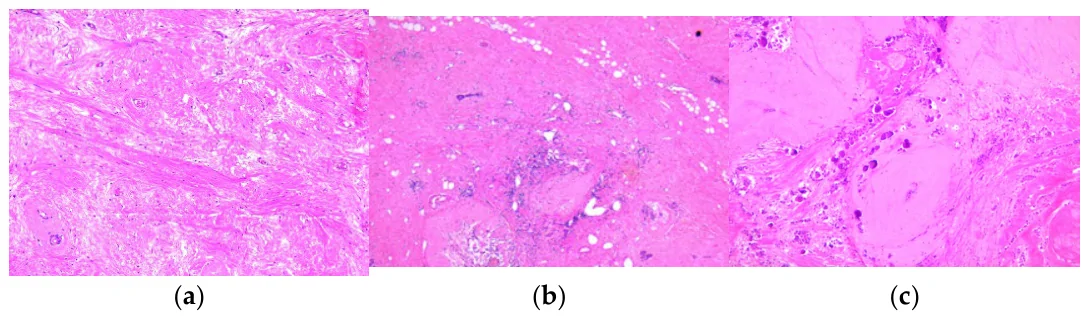
Microscopical features of the tumor bed with complete pathological response, ob 10×, H&E: (**a**) fibrosis, (**b**) inflammation, (**c**) calcifications.

**Figure 4:**
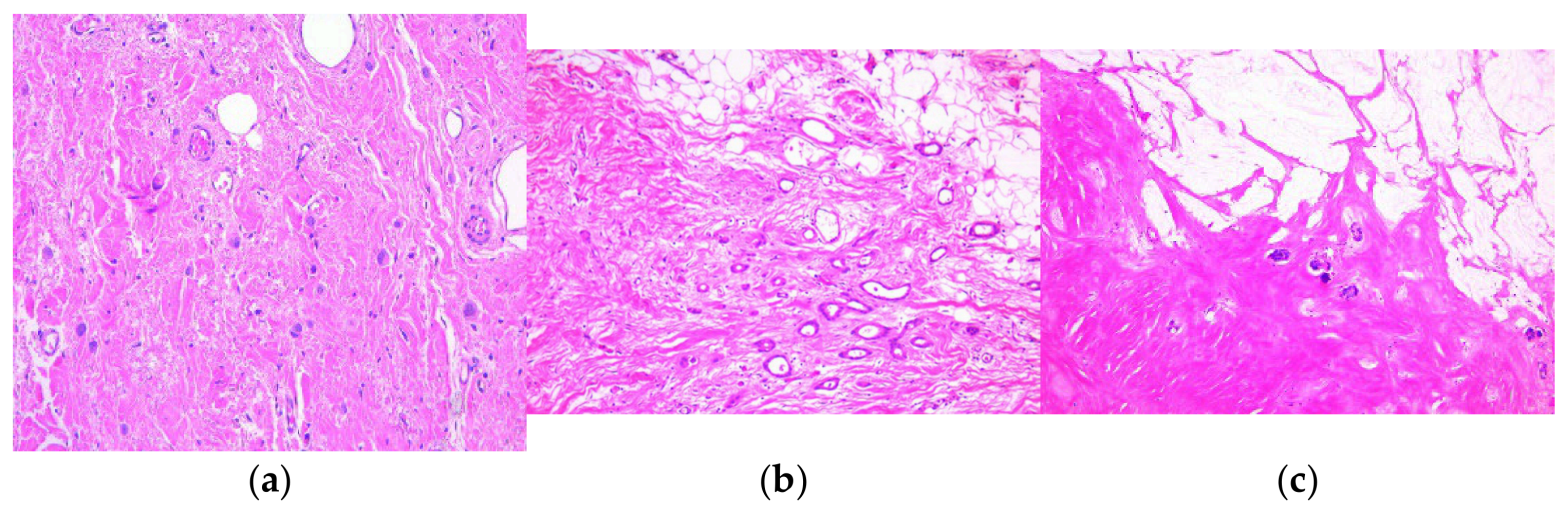
Microscopical features of the tumor bed with minimal residual disease, ob 10×, H&E: (**a**) isolated tumor cells, (**b**) tubule formation, (**c**) tumor nests and mucin lakes.

**Figure 5:**
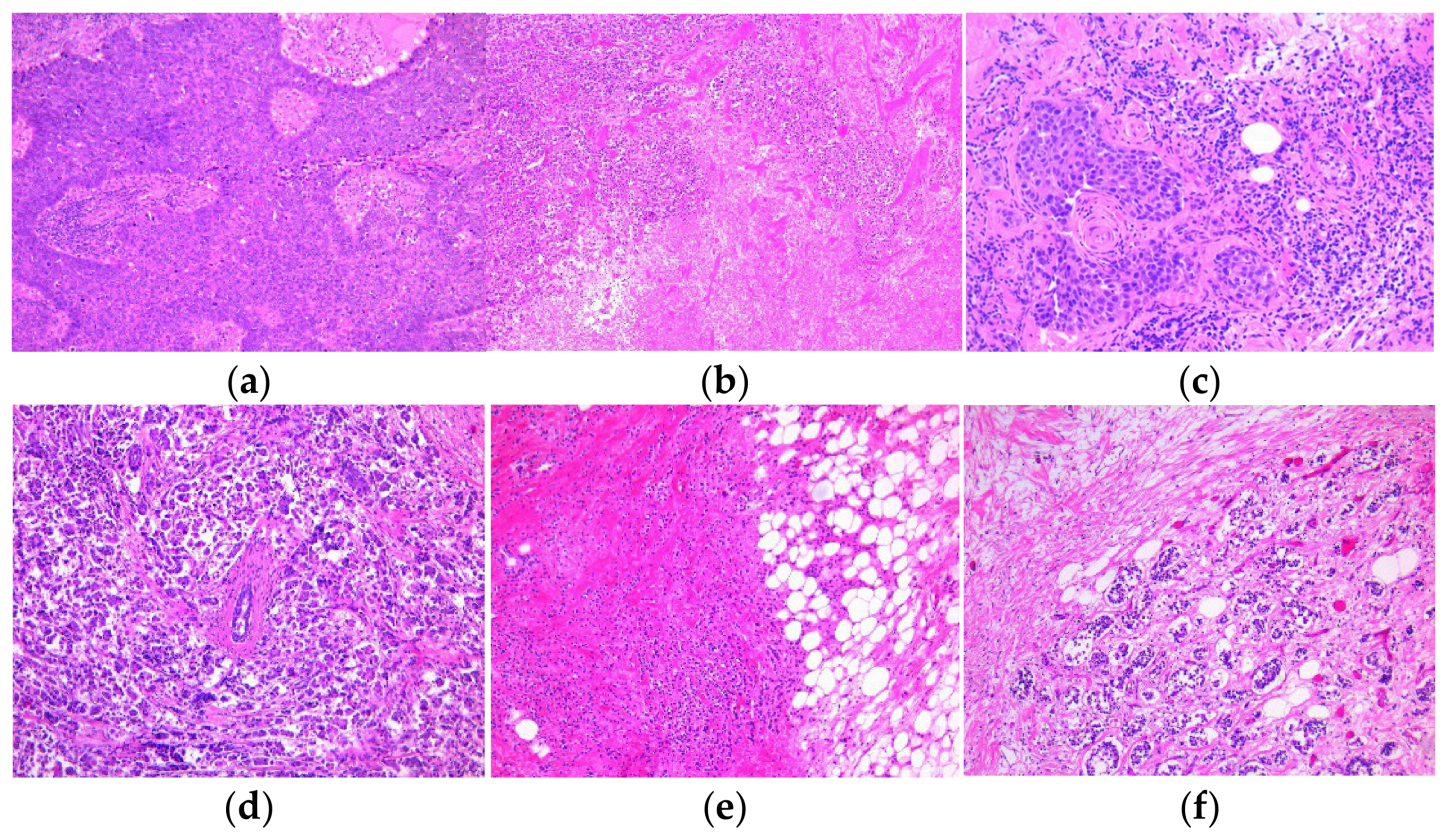
Microscopical features of the tumor bed with extensive residual disease, ob 10×, H&E: (**a**) solid growth, (**b**) tumor necrosis, (**c**) apocrine differentiation, (**d**) micropapillary growth, (**e**) lobular subtype, (**f**) nested growth.

The study cohort included 146 female patients treated with neoadjuvant therapy for breast carcinoma. All 146 patients were retained in the database and considered for descriptive analyses (Table 1). For each variable, the number of available observations was reported, and percentages were calculated using the number of non-missing observations as the denominator. Missing data were not imputed and were reported transparently for variables with incomplete information. Various morphological patterns of breast cancer on trucut biopsy are illustrated in Figure 2 and post-NAT tumor beds are illustrated in Figure 3, Figure 4 and Figure 5.

Post-treatment pathological (ypTNM) stage could be assigned in 142 of the 146 records and is summarized in Table 1. The most frequent pathological stage after neoadjuvant therapy was stage IIA, observed in 41/142 records (28.9%), followed by stage IA in 25/142 (17.6%), stage IIIA in 26/142 (18.3%), stage 0 in 20/142 (14.1%), stage IIB in 18/142 (12.7%), stage IIIB in 6/142 (4.2%) and stage IIIC in 6/142 (4.2%). Residual Cancer Burden could be calculated in 144 records: 20 (13.9%) were classified as RCB-0, 14 (9.7%) as RCB-I, 64 (44.4%) as RCB-II, and 46 (31.9%) as RCB-III. Overall, 110/144 records (76.4%) had RCB-II or RCB-III disease, indicating substantial residual tumor burden in most evaluable cases. Using the definition ypT0/is ypN0, pathological complete response was identified in 18/142 evaluable records (12.7%), while 124/142 (87.3%) were classified as non-pCR. Four records could not be classified for pCR because ypT and/or ypN status was unclear or unavailable. The case-by-case cross-tabulation of RCB class and pCR is shown in Table 2: all 18 pCR cases were RCB-0, and no pCR occurred in RCB-I, RCB-II or RCB-III; the two remaining RCB-0 records had no residual invasive carcinoma in the breast but an unevaluable ypN status due to the absence of an axillary specimen and were therefore conservatively not counted as pCR, whereas the other two pCR-unclassifiable records were also not evaluable for RCB. Thus RCB-0 and pCR were fully concordant in all records in which both could be determined.

Paired pre- and post-treatment comparisons showed no significant change in stromal TILs, which had a median of 3% (IQR 2–8%) before treatment and 3% (IQR 2–10%) after treatment (*p* = 0.813). Tumor cellularity showed a significant reduction, decreasing from a median of 70% (IQR 50–80%) before therapy to 50% (IQR 10–80%) after therapy, with a median paired change of −10 percentage points (*p* < 0.001). Tumor grade did not differ significantly between pre- and post-treatment specimens among evaluable paired records (*p* = 0.308). Tumor necrosis decreased from 27/146 records (18.5%) before treatment to 17/146 (11.6%) after treatment, but this difference was not statistically significant (*p* = 0.123). The in situ component was recorded more frequently after therapy, increasing from 29/146 records (19.9%) to 50/146 (34.2%; *p* = 0.004). Vascular invasion was also recorded more frequently after therapy, increasing from 34/146 records (23.3%) to 75/146 (51.4%; *p* < 0.001). Perineural invasion was present in 6/146 records (4.1%) both before and after treatment; however, the six cases were not the same in the two specimens (paired 2 × 2 table: present in both specimens, 0; present only in the biopsy, 6; present only in the resection specimen, 6; absent in both, 134), giving a McNemar chi-square statistic with continuity correction of (|6 − 6| − 1)^2^/(6 + 6) = 0.083 (*p* = 0.773) and an exact binomial McNemar *p* = 1.000.

Baseline predictors of response were explored by comparing patients with good pathological response, defined as RCB 0/I, with those with poor pathological response, defined as RCB II/III. Overall, 34 patients were classified as RCB 0/I and 110 as RCB II/III. Age did not differ significantly between response groups, with a median age of 63.5 years (IQR 55–70.8) in the RCB 0/I group and 65 years (IQR 55.2–71) in the RCB II/III group (*p* = 0.585). Baseline tumor grade (*p* = 0.435) and tumor cellularity (median 72.5% versus 70%; *p* = 0.779) were not associated with response. Median baseline sTILs were higher among good responders than among poor responders (5% [IQR 2–12.5] versus 3% [IQR 2–7]), a difference in borderline significance (*p* = 0.069), and the distribution of sTILs categories differed between groups (intermediate or high sTILs in 7/28 [25.0%] good responders versus 11/103 [10.7%] poor responders; *p* = 0.043). Baseline histological type was not associated with response (*p* = 0.390). In contrast, the distribution of baseline surrogate subtypes differed significantly between response groups (*p* = 0.001): among patients with RCB 0/I, 9/34 (26.5%) were HER2-positive, 8/34 (23.5%) triple-negative, 14/34 (41.2%) Luminal B and only 1/34 (2.9%) Luminal A, compared with 9/110 (8.2%), 12/110 (10.9%), 59/110 (53.6%) and 25/110 (22.7%), respectively, in the RCB II/III group. A baseline in situ component (3/34 [8.8%] versus 25/109 [22.9%]; *p* = 0.085) and lymphovascular invasion (4/34 [11.8%] versus 30/108 [27.8%]; *p* = 0.067) were less frequent among good responders, without reaching statistical significance. Baseline necrosis (*p* = 0.805) and perineural invasion (*p* = 0.336) were not associated with response. Given the modest number of patients with RCB 0/I response, these analyses should be interpreted as exploratory.

Post-treatment molecular subtype could be classified or explicitly designated unclassified in 58 records. Among the 57 records with both an unambiguous baseline subtype and an evaluable post-treatment classification, 23 (40.4%) retained the same subtype and 34 (59.6%) showed a different post-treatment classification. Among baseline Luminal A tumors, 7/12 remained Luminal A, 3/12 were reclassified as Luminal B, and 2/12 as triple-negative. Among baseline Luminal B tumors, 11/27 remained Luminal B, 14/27 were reclassified as Luminal A, 1/27 as mixed/ambiguous, and 1/27 as unclassified. Of the baseline triple-negative tumors, 3/8 remained triple-negative, 3/8 were reclassified as Luminal A, and 2/8 became unclassified. Among baseline HER2-positive tumors, 2/10 remained HER2-positive, 2/10 were reclassified as Luminal A, 4/10 as Luminal B, 1/10 as triple-negative, and 1/10 as unclassified. One additional record had a mixed/ambiguous baseline subtype and was excluded from the stability calculation. Importantly, not all of these 34 changes represent biological conversion of receptor status: 5/57 (8.8%) were reclassified only because the post-treatment profile was unclassified (n = 4) or mixed/ambiguous (n = 1), and 17/57 (29.8%) involved a shift between Luminal A and Luminal B, which is driven by a change in Ki-67 and/or PR expression across the classification threshold rather than by loss or gain of ER or HER2. A change in ER or HER2 status category was observed in 12/57 records (21.1%): Luminal A to triple-negative (n = 2), triple-negative to Luminal A (n = 3), and HER2-positive to Luminal A, Luminal B or triple-negative (n = 7). Marker-by-marker changes in ER, PR, HER2 and Ki-67 could not be analyzed separately because the post-treatment database recorded the resulting surrogate subtype rather than the individual marker results. Given that post-treatment molecular data were available only for a subset of records, this analysis was considered exploratory.

**Figure 6:**
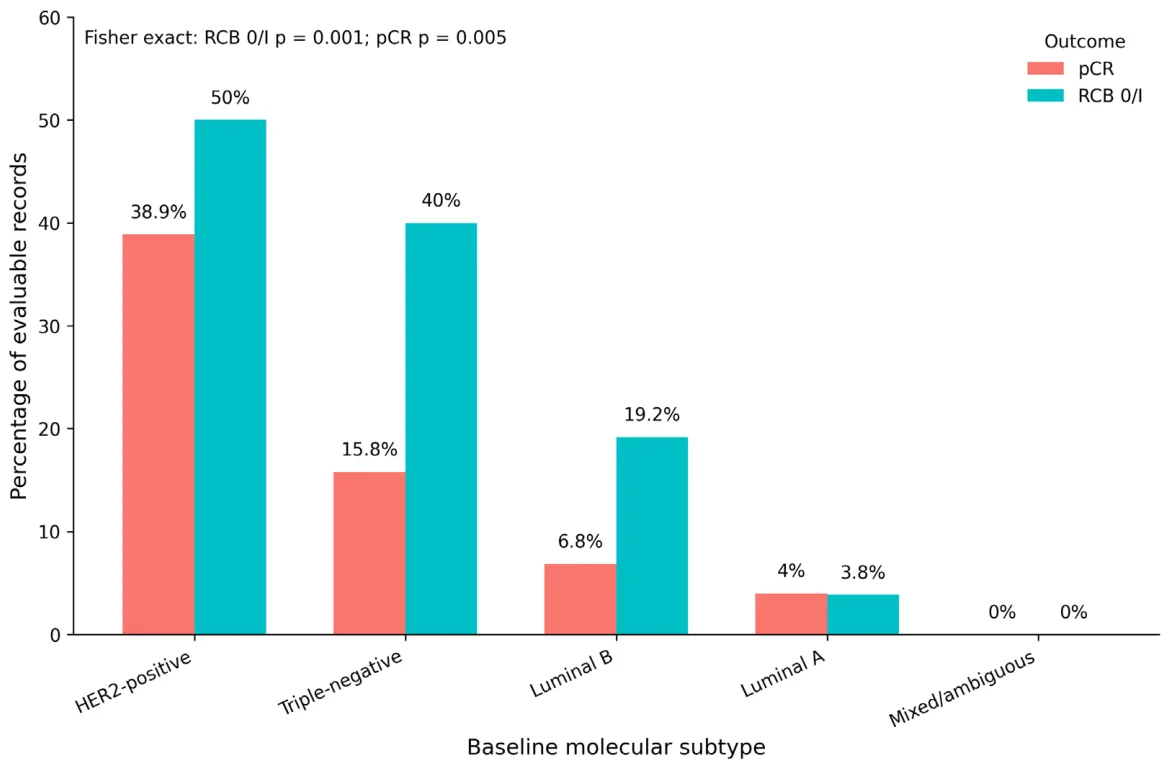
Pathological response according to baseline molecular subtype.

Baseline molecular subtype was significantly associated with pathological response (Figure 6). When response was categorized as RCB 0/I versus RCB II/III, the distribution of response differed significantly across molecular subtypes (*p* = 0.001). Good pathological response was observed in 9/18 HER2-positive tumors (50.0%), 8/20 triple-negative tumors (40.0%), 14/73 Luminal B tumors (19.2%), and 1/26 Luminal A tumors (3.8%); neither of the two mixed/ambiguous tumors achieved RCB 0/I. A similar pattern was observed for pathological complete response. Baseline molecular subtype was significantly associated with pCR (*p* = 0.005), with pCR occurring in 7/18 HER2-positive tumors (38.9%), 3/19 triple-negative tumors (15.8%), 5/73 Luminal B tumors (6.8%), and 1/25 Luminal A tumors (4.0%); no pCR occurred among the two mixed/ambiguous tumors. These findings should be interpreted cautiously because some molecular subgroups remained small and outcome denominators varied according to data availability.

**Figure 7:**
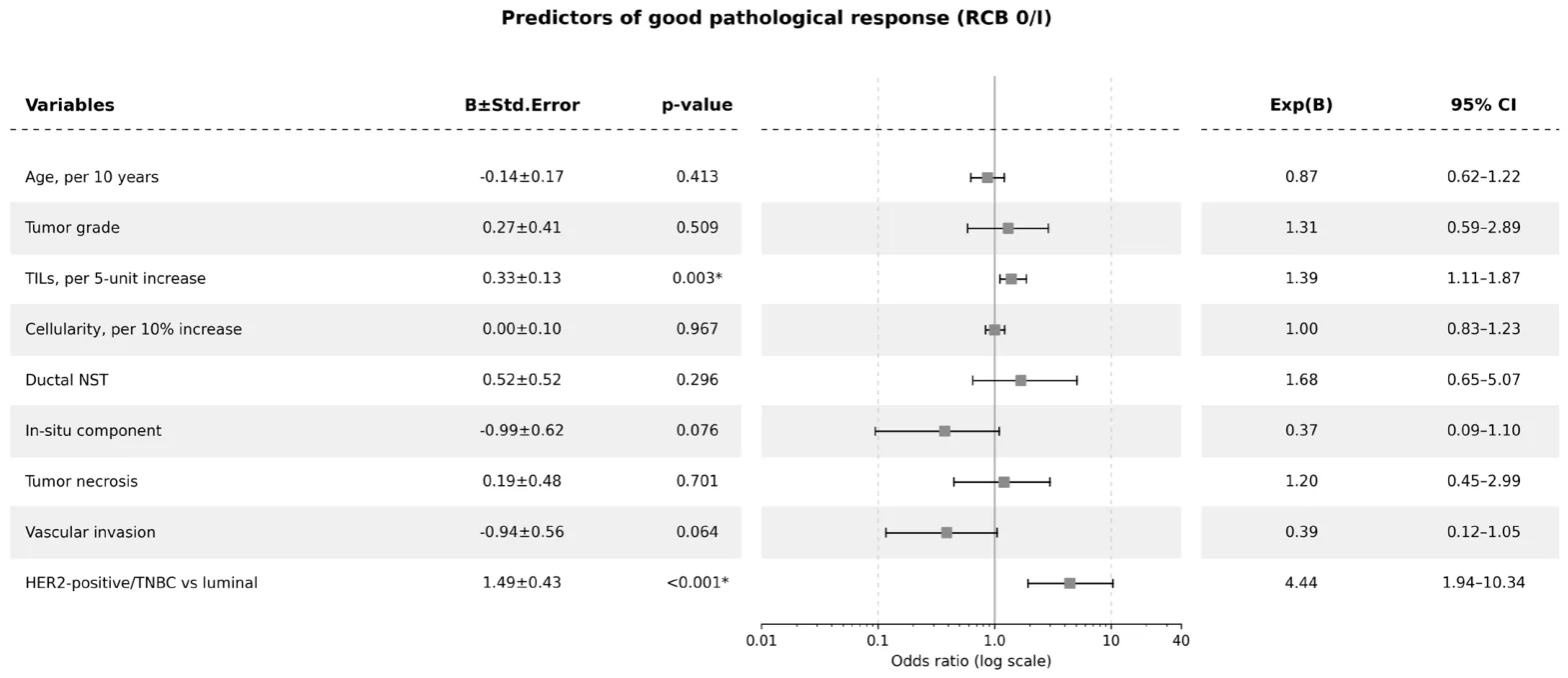
Univariable Firth penalized logistic regression analysis for favorable pathological response, defined as RCB-0/I.

**Figure 8:**
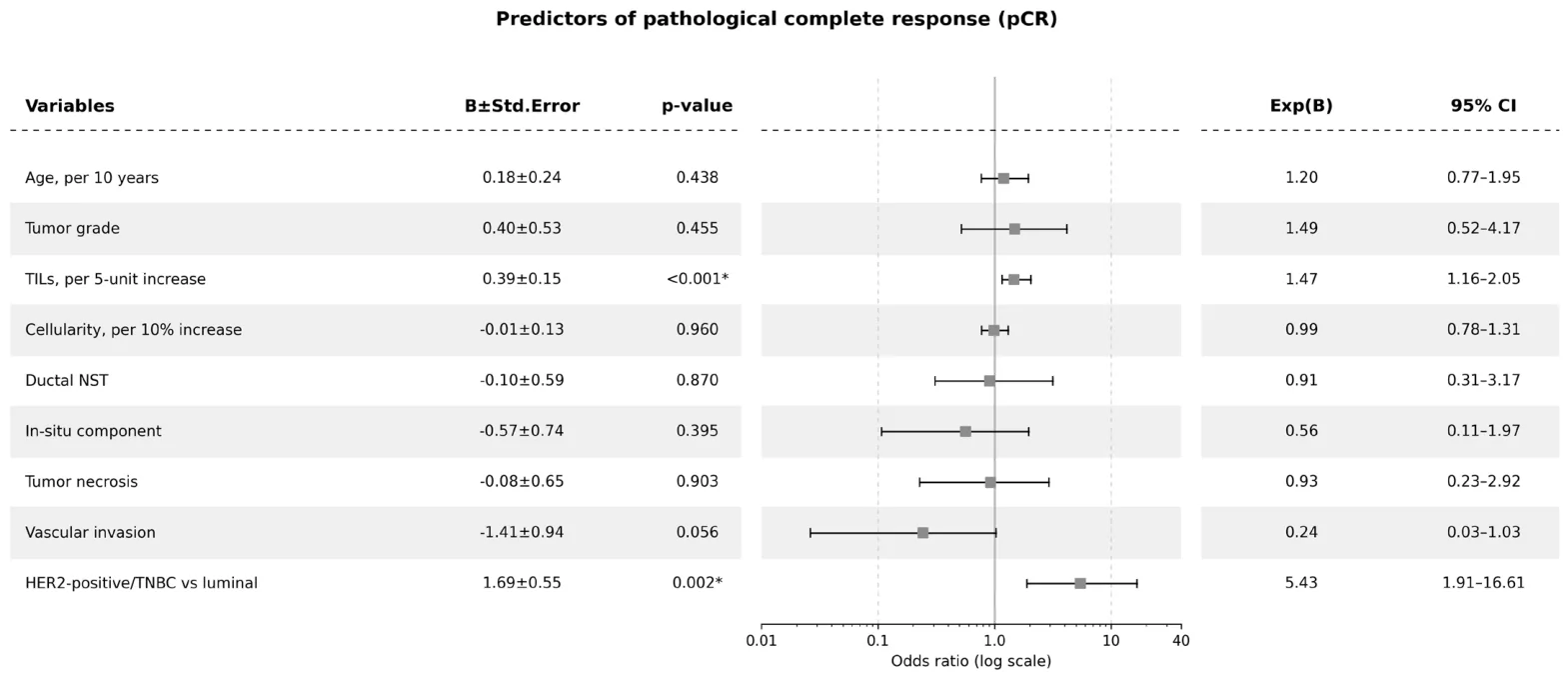
Univariable Firth penalized logistic regression for pathological complete response, defined as ypT0/is ypN0. Odds ratios are shown on a logarithmic scale.

Because the number of favorable pathological responses remained limited, predictors of response were further explored using univariable Firth penalized logistic regression (Figure 7 and Figure 8). For good pathological response, defined as RCB 0/I, higher baseline TILs were significantly associated with increased odds of response, with an odds ratio of 1.39 per 5-percentage-point increase (95% CI 1.11–1.87; *p* = 0.003). HER2-positive or triple-negative tumors also had higher odds of achieving RCB 0/I than luminal tumors (OR = 4.44, 95% CI 1.94–10.34; *p* < 0.001). The presence of an in situ component and vascular invasion before therapy showed non-significant inverse trends (OR = 0.37, *p* = 0.076 and OR = 0.39, *p* = 0.064, respectively). For pathological complete response, higher baseline TILs remained significantly associated with pCR (OR = 1.47 per 5-percentage-point increase, 95% CI 1.16–2.05; *p* < 0.001), and HER2-positive or triple-negative tumors had higher odds of pCR than luminal tumors (OR = 5.43, 95% CI 1.91–16.61; *p* = 0.002). Baseline vascular invasion showed a borderline inverse association with pCR (OR = 0.24, 95% CI 0.03–1.03; *p* = 0.056). Age, grade, tumor cellularity, histological group, in situ component, and tumor necrosis were not significantly associated with pCR. Overall, these univariable analyses identify baseline TILs and molecular subtype as the most relevant exploratory predictors of pathological response.

**Figure 9:**
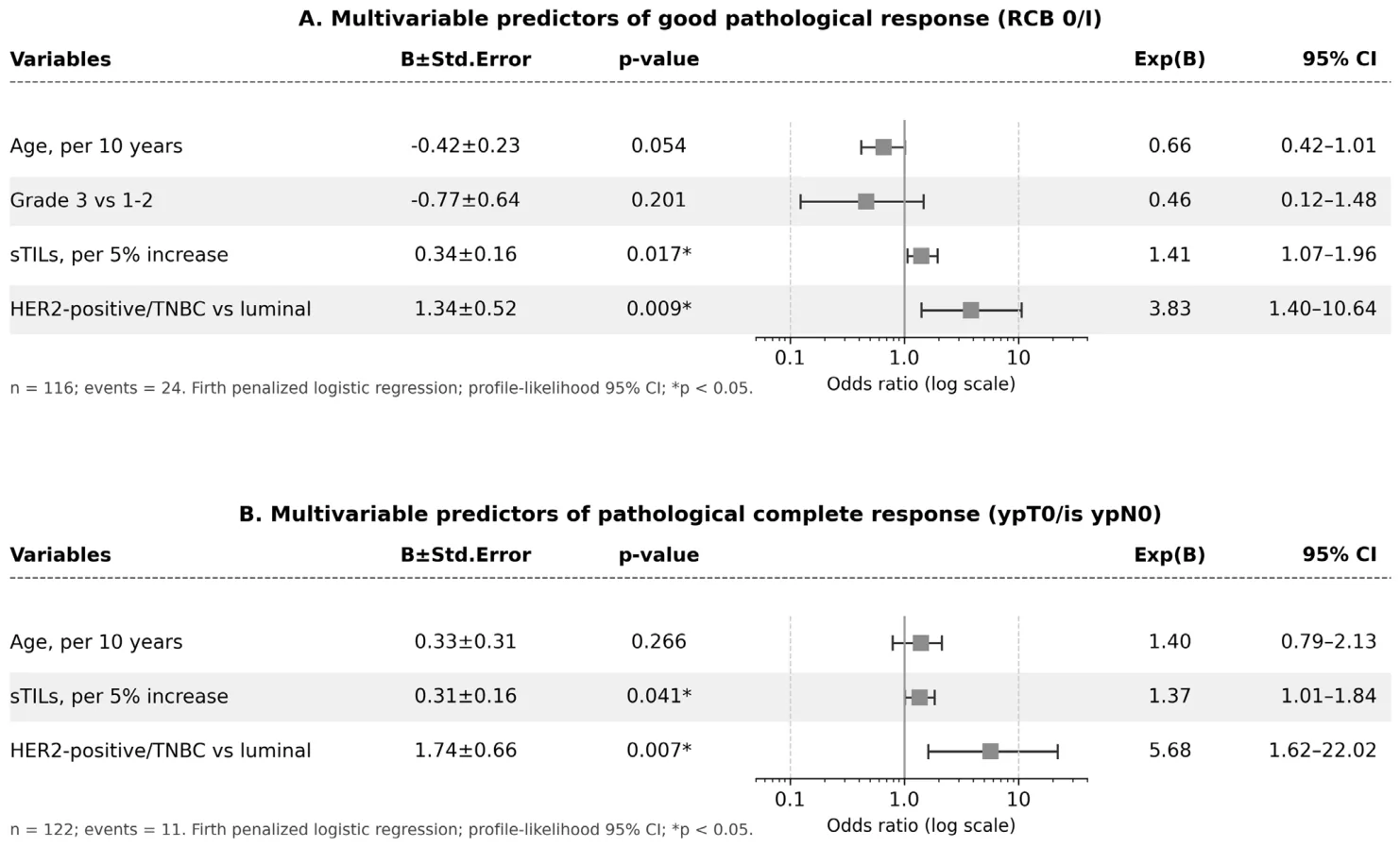
Multivariable Firth penalized logistic regression for (**A**) favorable pathological response, defined as RCB 0/I, and (**B**) pathological complete response, defined as ypT0/is ypN0. Odds ratios are shown on a logarithmic scale with profile-likelihood 95% confidence intervals; n and the number of events refer to complete-case analyses.

In multivariable Firth regression (Figure 9), higher baseline sTILs (OR = 1.41 per 5-percentage-point increase, 95% CI 1.07–1.96; *p* = 0.017) and HER2-positive/triple-negative subtype (OR = 3.83, 95% CI 1.40–10.64; *p* = 0.009) remained associated with RCB 0/I after adjustment for age and Nottingham grade, whereas older age showed a borderline inverse association (OR = 0.66 per 10 years, 95% CI 0.42–1.01; *p* = 0.054) and grade 3 was not independently associated with response (OR = 0.46, 95% CI 0.12–1.48; *p* = 0.201). Adding the baseline in situ component and lymphovascular invasion to the model did not materially change these estimates (sTILs: OR = 1.38, 95% CI 1.03–1.92; HER2-positive/triple-negative: OR = 3.39, 95% CI 1.23–9.42), and neither variable was independently associated with response (OR = 0.52, 95% CI 0.09–1.99 and OR = 0.89, 95% CI 0.23–2.94, respectively). For pCR, in a model limited to three covariates because of the number of events, baseline sTILs (OR = 1.37 per 5-percentage-point increase, 95% CI 1.01–1.84; *p* = 0.041) and HER2-positive/triple-negative subtype (OR = 5.68, 95% CI 1.62–22.02; *p* = 0.007) were independently associated with pCR, whereas age was not (OR = 1.40 per 10 years, 95% CI 0.79–2.13; *p* = 0.266). Because complete data on all covariates were available for 116 patients (24 RCB 0/I events) and 122 patients (11 pCR events), the multivariable estimates are less precise than the univariable ones and should be regarded as exploratory.

**Figure 10:**
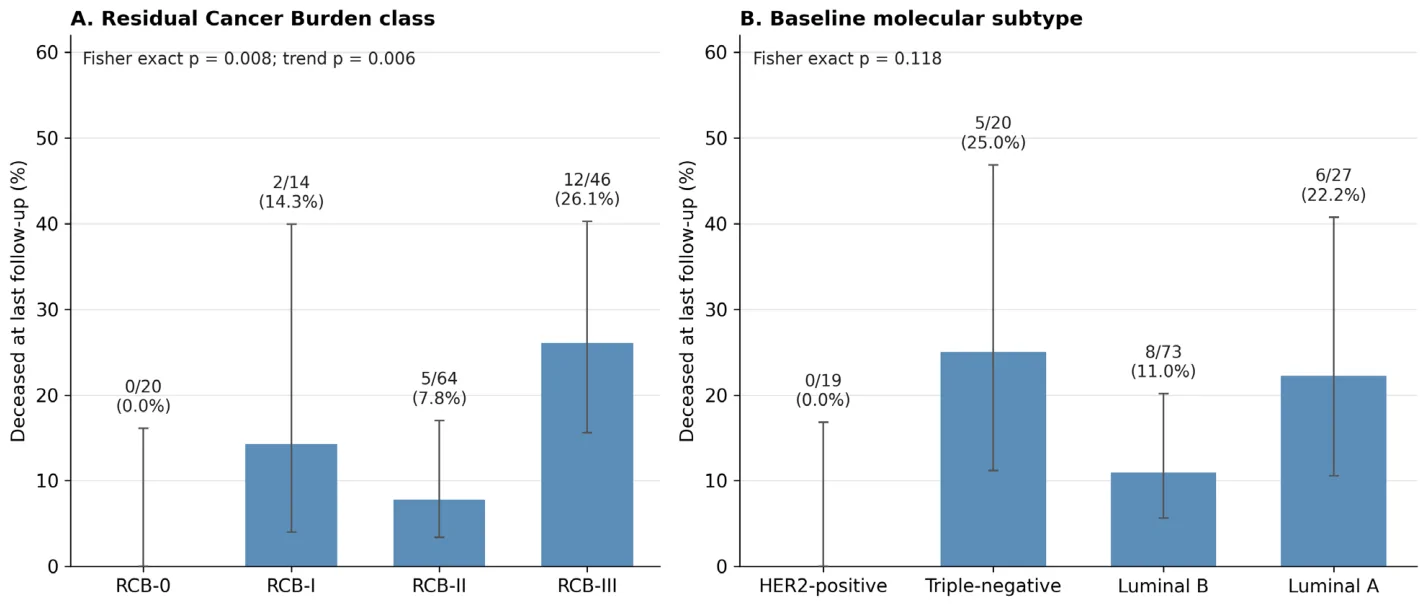
Crude mortality according to (**A**) Residual Cancer Burden class and (**B**) baseline surrogate molecular subtype. Bars show the proportion of deceased patients with Wilson 95% confidence intervals; numbers above the bars indicate deaths/patients.

Nineteen of the 146 patients (13.0%) were deceased at time of the last follow-up; the date of death was documented for 17 of them (between November 2021 and June 2026) and the interval from surgery to death was available for seven patients (median 2.0 years, range 0.5–4.0). Crude mortality according to pathological response and baseline features is summarized in Table 3. Mortality differed significantly across RCB classes (Figure 10A): no death occurred among the 20 patients with RCB-0, whereas 2/14 (14.3%) patients with RCB-I, 5/64 (7.8%) with RCB-II and 12/46 (26.1%) with RCB-III had died (Fisher’s exact *p* = 0.008; *p* for trend = 0.006), and in univariable Firth regression each increase in RCB class was associated with higher odds of death (OR = 2.52 per class, 95% CI 1.32–5.69; *p* = 0.003). When response was dichotomized, mortality was 2/34 (5.9%) in the RCB 0/I group versus 17/110 (15.5%) in the RCB II/III group (*p* = 0.245; OR = 2.43, 95% CI 0.71–12.69), and no death was recorded among the 18 patients who achieved pCR, compared with 19/124 (15.3%) non-pCR patients (*p* = 0.131; OR = 0.15, 95% CI 0.00–1.16; *p* = 0.075). Mortality did not differ significantly according to baseline surrogate subtype (Figure 10B): 0/19 HER2-positive, 5/20 (25.0%) triple-negative, 8/73 (11.0%) Luminal B and 6/27 (22.2%) Luminal A patients had died (*p* = 0.118). Baseline tumor necrosis was associated with higher mortality (7/27 [25.9%] versus 12/117 [10.3%]; *p* = 0.052; OR = 3.09, 95% CI 1.07–8.49; *p* = 0.037), whereas age (*p* = 0.963), grade (*p* = 0.203), histological type (*p* = 0.819), lymphovascular invasion (*p* = 0.392) and the in situ component (*p* = 1.000) were not. Baseline sTILs were slightly higher among deceased patients (median 5% versus 3%; *p* = 0.003), reflecting the higher sTILs of triple-negative tumors, which accounted for five of the 19 deaths; sTILs modeled as a continuous variable (OR = 1.10 per 5 percentage points, 95% CI 0.86–1.33; *p* = 0.391) and sTILs category (*p* = 1.000) were not associated with death. Post-treatment tumor cellularity was higher in deceased than in surviving patients (median 72.5% versus 40%; *p* = 0.036), consistent with the association between residual burden and death. In a multivariable Firth model including age and surrogate subtype, RCB class remained associated with death (OR = 2.68 per class, 95% CI 1.37–6.15; *p* = 0.003), whereas age (OR = 0.91 per 10 years, 95% CI 0.58–1.48) and HER2-positive/triple-negative subtype (OR = 1.53, 95% CI 0.47–4.57) were not.

## 4. Discussion

Breast cancer is the most common malignancy in women worldwide. However, due to advances in systemic treatments that are now standard care, 70–80% of patients with early-stage, non-metastatic disease can be cured [27,28]. This study evaluated a cohort of 146 women diagnosed with breast cancer at our institution who received neoadjuvant therapy followed by surgical intervention (either mastectomy or breast-conserving surgery).

Most patients were diagnosed via core needle biopsy, which is the gold standard for evaluating lesions detected clinically or radiologically [29]. All patients underwent neoadjuvant treatment—consisting of chemotherapy, endocrine therapy, HER2-targeted therapy, or combinations thereof—in compliance with the NCCN guidelines. Following systemic therapy, 83.6% of patients underwent mastectomy with axillary dissection, while 14.4% had sectorectomies (breast-conserving surgery) with axillary dissection. Because treatment allocation is itself subtype-driven, the type of neoadjuvant treatment and the biological subtype are confounded by design in a real-world cohort such as ours: HER2-positive tumors receive chemotherapy plus HER2-targeted therapy, triple-negative tumors receive intensified chemotherapy (with the addition of immune checkpoint inhibition in current guidelines), whereas some hormone receptor-positive tumors receive endocrine therapy alone, which is known to produce pCR only exceptionally. The higher response rates observed in HER2-positive and triple-negative tumors should therefore be interpreted as the response of each subtype under its guideline-recommended treatment.

Different parameters evaluated on the biopsy and surgical specimens were correlated with the response to therapy according to the M.D. Anderson RCB staging system. Residual Cancer Burden was available for 144 of the 146 patients, with the majority of patients with RCB-II or RCB-III disease, indicating the persistence of substantial residual tumor burden in most cases.

In alignment with historical literature, the two most common histological types identified were invasive breast carcinoma of no special type (NST) [30], which accounted for 77.4% of the cases in our study, followed by invasive lobular carcinoma. Histological grade correlates strongly with patient prognosis [31] and it is based on the Nottingham score calculated for each biopsy. The majority of the analyzed tumors were classified as moderately differentiated. However, this percentage may be influenced by the inherent subjectivity involved in assessing both quantitative features (the percentage of tubule formation and mitotic count) and qualitative features (nuclear pleomorphism).

Histologic grade is also strongly associated with both breast cancer-specific survival and disease-free survival [32] independent of NAT. In our study we wanted to compare tumor grading before and after neoadjuvant therapy for tumors with complete or partial pathological response. The continuous RCB score is categorized into four prognostically distinct classes: RCB-0 (pCR) indicates no residual invasive disease with excellent prognosis across subtypes; RCB-I (minimal residual disease) shows favorable prognosis; RCB-II (moderate residual disease) represents intermediate prognosis and is the most common category; and RCB-III (extensive residual disease) indicates poor prognosis [33]. A good pathological response is defined as RCB 0/I, while a poor pathological response is defined as RCB II/III. RCB I and RCB II define a partial pathological response. Tumor grade did not differ significantly between pre- and post-treatment specimens. More than 70% of the tumors with partial response were moderately and well differentiated, a finding supporting that Nottingham score is not only a strong prognosis factor for breast carcinomas in general, but also a predictive factor for tumor response to NAT. Among the tumors that achieved a complete or partial response following NAT, only two exhibited a higher histological grade, while the remainder demonstrated an equal or lower grade. This observation suggests that a comparative assessment of tumor grade, with its preservation or downgrading after NAT, may deserve evaluation as a prognostic factor in larger series with survival follow-up.

When it comes to the two types of tumor infiltrating lymphocytes, intratumoral TILs are defined as lymphocytes in tumor nests and stromal TILs, located dispersed in the stroma between the tumor cells [34]. The latter are considered to be a prognostic biomarker, predictive of response to immunotherapy, chemotherapy, and other targeted therapies [35]. There are studies like the ones conducted by the German Breast Cancer Group reporting that increased TILs concentration on the initial biopsy predicted response to neoadjuvant chemotherapy in all molecular subtypes assessed, and was also associated with a survival benefit in HER2-positive breast cancer and TNBC [36]. In our cohort, however, baseline sTILs were not associated with crude mortality, and the small number of deaths precluded a meaningful subtype-specific analysis of this question. In our study, the majority of tumors were in the low sTILs group and sTILs did not change significantly after neoadjuvant therapy, remaining within the low category. Median baseline sTILs were higher among good responders than among poor responders (5% versus 3%); a difference in borderline significance and higher baseline sTILs were associated with increased odds of RCB 0/I in both the univariable and the multivariable Firth models, independently of age, grade and surrogate subtype. For complete pathological response, higher baseline TILs remained significantly associated with pCR, consistent with numerous other studies including a meta-analysis of 3251 patients in 13 published studies [37]. Given this difference in the distribution of inflammatory cells before and after NAT, we support the reporting of TILs also in the post-neoadjuvant setting, as it may be relevant in large-scale studies to predict the benefit of some chemotherapeutic and targeted agents [38].

Residual tumor cellularity is a very important prognostic factor associated with improved outcomes in breast cancer [39] and a key component of the RCB score. In the study that we conducted, tumor cellularity showed a significant reduction after NAT, and residual (post-treatment) cellularity was higher among patients who died, in keeping with its prognostic role as a component of the RCB score. There are studies such as the one conducted by Chic et al. [40] trying to introduce a novel biomarker based on the combination of stromal tumor-infiltrating lymphocytes and tumor cellularity for HER2-positive tumors assessed at 2 weeks post-therapy. Other studies like Sagili et al. [41] evaluated residual tumor cellularity correlated with residual tumor size in locally advanced breast cancer.

Breast cancer is a genetically and clinically heterogeneous disease [42]. It is well-known that certain special types of breast cancer like metaplastic and invasive lobular carcinoma have a significant worse prognosis [43]. In our cohort, baseline histological type was not associated with response group. Tumors sharing the same surrogate subtype may differ in intrinsic molecular subtype, genomic drivers and germline background; in triple-negative breast cancer, for instance, pathogenic germline variants, predominantly in BRCA1, are identified in a substantial proportion of patients and are associated with distinct clinicopathological features [43], and such underlying genetic heterogeneity may contribute to the wide spectrum of response observed within this subtype in our cohort, where RCB 0/I was achieved by 40% of triple-negative tumors while the remaining 60% retained moderate or extensive residual disease. Similarly, the luminal B category encompasses tumors with a broad range of proliferation and HER2 expression, and the frequent post-treatment change in surrogate subtype that we observed illustrates the phenotypic plasticity of these tumors and the spatial and temporal heterogeneity of receptor expression [37,44]. Larger cohorts with molecular profiling would be required to determine whether the associations reported here hold within molecularly defined subgroups.

Tumor necrosis is another important variable in biopsy reporting, with adverse prognostic significance [45], associated with molecular features like ER and PR negativity, HER2 positivity, and the basal-like subtype of breast cancer [46]. In line with the evolving characterization of other pathological variables, the definition and prognostic value of necrosis have been significantly refined. Current literature indicates that confluent tumor necrosis (TN) of any dimension within the invasive regions of node-positive breast cancer serves as an independent predictor of early recurrence and disease-specific mortality [44]. It is believed that necrotic cells enhance angiogenesis and proliferation of endothelial cells, induce vasculature, as well as increase migration, invasion, and cell–cell interactions [47]. We evaluated tumor necrosis on the initial biopsy and following NAT and observed a numerical, although not statistically significant, decrease after therapy (18.6% versus 11.7%; *p* = 0.123). Notably, baseline necrosis was associated with higher crude mortality in our cohort, in keeping with its reported adverse prognostic significance [48], although this observation is based on a small number of deaths.

Carcinoma in situ is a nonobligate precursor of invasive cancer [49] and an important variable in determining the RCB score. The general belief is that there is no association between residual CIS and recurrence after neoadjuvant chemotherapy, thus pCR does not take into consideration the presence of CIS [50].

Additionally, at baseline, angiolymphatic invasion was observed in about one-quarter of the tumors, while about one-fifth exhibited necrosis or carcinoma in situ. Both the in situ component and lymphovascular invasion were recorded more frequently in the resection specimens than in the pretreatment biopsies. This difference should not be interpreted as a therapy-induced increase: a core-needle or incisional biopsy samples only a small fraction of the tumor, whereas the resection specimen allows examination of the entire tumor bed, its periphery and the peritumoral vessels, so that the higher post-treatment detection rates most likely reflect the difference in sampling extent between the two specimen types (and, for lymphovascular invasion, the tendency of residual tumor to persist within vascular spaces after therapy). These paired comparisons are therefore descriptive, and the biopsy-based frequencies should be regarded as minimum estimates.

It is well known that molecular heterogeneity exists in breast cancer as varying expression of the ER, PR, and HER2 [51] as the result of genetic and non-genetic alterations [52]. Inter-tumor heterogeneity can be correlated with molecular alterations as is the case of BRCA mutations in triple negative breast cancer [53]. Intratumoral heterogeneity before and after therapy is an important limitation in the evaluation of the response to therapy and is driven by both intrinsic effects of the tumor cells themselves as well as extrinsic effects from the surrounding microenvironment [54]. Invasive cancer subtypes such as the luminal A and B subtypes show better prognosis and response to hormone receptor-targeted therapies compared with the triple-negative subtypes; on the other hand, triple-negative tumors respond better than luminal tumors to chemotherapy [55]. Our findings align with these observations, as the baseline molecular subtype was significantly associated with pathological response. The highest proportion of tumors achieving a favorable pathological response was observed in the HER2-positive category, followed by triple-negative and Luminal B subtypes. In contrast, only one Luminal A tumor (3.8%) achieved an RCB 0/I response. A matching pattern was observed regarding pCR.

Molecular subtypes in breast cancer frequently alter following neoadjuvant chemotherapy, which directly impacts tumor prognostication. Consequently, molecular subtyping should be repeated on the residual surgical specimen after NAT [56]. Part of this shifting profile may be driven by therapy-induced phenotypic adaptation, wherein systemic treatment alters or downregulates gene expression, for instance through epigenetic silencing of receptors, or by selection of pre-existing clones with a different receptor profile. In our study, more than half of the tumors retested in our laboratory showed a different post-treatment classification; however, as detailed in the Results, only about one-fifth of the retested tumors showed a change in ER or HER2 status category, whereas the remaining changes consisted of shifts between Luminal A and Luminal B driven by Ki-67 and/or PR, or of post-treatment profiles that were unclassifiable or mixed. The latter cannot be counted as true biological conversion and should not be attributed to epigenetic silencing. Moreover, discordance in receptor status between paired specimens should also take the following testing and interpretation factors into account: biopsy and resection specimens differ in fixation time and tissue volume, retesting was performed at different time points rather than in a single batch, Ki-67 and PR are subject to considerable inter-observer and inter-run variability near the classification thresholds, and HER2 interpretation itself may be technically difficult under particular chromosomal backgrounds, as illustrated by cases with complete deletion of the chromosome 17 centromere (CEP17), in which the standard HER2/CEP17 ratio cannot be calculated and alternative chromosome 17 control probes or cytogenomic microarray may be required to resolve HER2 status [57]. Our findings nevertheless support the practice of retesting receptor status on residual tumors, recurrent tumors or metastases, ideally with paired specimens stained in the same batch and with in situ hybridization for equivocal HER2 results. A downside of this statistic is the availability of the data only in a subset of patients.

In the multivariable Firth models, baseline stromal tumor-infiltrating lymphocytes (sTILs) and surrogate molecular subtype were associated with pathological response independently of age and Nottingham grade; although adjustment for treatment regimen and clinical stage was not possible, these results support the value of both variables as exploratory predictors of response. In the future, such findings could be combined with established data showing that Ki67 expression and lymphovascular invasion act as independent risk factors for extensive post-treatment residual disease [58]. Furthermore, Residual Cancer Burden (RCB) scoring independently determines prognosis across all breast cancer subtypes [50], where achieving a pathological complete response (pCR) remains fundamentally linked to superior event-free and overall survival outcomes [25]. Our vital-status analysis is consistent with these data: although survival could not be estimated, crude mortality increased across RCB classes, no death was recorded among patients with RCB-0 or pCR, and RCB class remained associated with death after adjustment for age and subtype.

It is well established that luminal breast cancers, and in particular Luminal A tumors, show the lowest rates of pathological complete response to neoadjuvant chemotherapy, whereas HER2-positive and triple-negative tumors respond best, and our results confirm this pattern. The contribution of the present study does not lie in this confirmation but in several aspects that are less frequently reported. First, the same panel of routine histopathological variables (histological type, grade, sTILs, tumor cellularity, necrosis, in situ component, lymphovascular and perineural invasion and surrogate subtype) was assessed in a paired fashion on the pretreatment biopsy and on the resection specimen of the same patients, allowing a direct description of therapy-associated changes and of the limitations of biopsy-based assessment. Second, the cohort is an unselected, real-world series from an Eastern European center, treated outside clinical trials and including patients who received neoadjuvant endocrine therapy, a setting in which RCB- and sTILs-based data remain scarce. Third, baseline sTILs retained an association with RCB 0/I and pCR independently of surrogate subtype, age and grade even in a cohort in which sTILs were predominantly low, suggesting that the predictive information of sTILs is not restricted to the high-sTILs tumors described in trial populations. Fourth, we quantified the frequency and the nature of post-treatment subtype reclassification and showed that only a minority of changes involve ER or HER2 status. Finally, the exploratory vital-status analysis links RCB class and baseline tumor necrosis to crude mortality in this population. These elements, rather than the subtype–response association itself, are the findings that we consider worth validating in larger, treatment-stratified cohorts.

Treatment in breast tumors relies on the additional detection, in selected cases, of unimolecular alterations like phosphatidylinositol-4,5-bisphosphate 3-kinase catalytic subunit alpha (PIK3CA) and estrogen receptor 1 (ESR1) in hormone-sensitive cases, *BRCA* gene mutations in HER2-negative cancers, and programmed cell death-1 ligand (PD-L1) in triple-negative breast cancer (TNBC) [48]. Tumor-infiltrating lymphocytes (TILs) are part of the newer, emerging biomarkers with predictive and possibly therapeutic value in breast cancer [59]. Precision oncology recognizes that cancer is a highly heterogeneous disease with a distinct genetic diversity for each tumor [60]. The newest research is focused on genetic, epigenetic, proteomic, and metabolomic markers with diagnostic, prognostic and therapeutic implications for individualized treatment strategies [61]. Liquid biopsy is an alternative to tissue biopsy that offers the opportunity to conduct repeated sampling and analysis of biological samples to evaluate biomarker expression in malignant breast tumors at different disease stages [62] and monitor tumor heterogeneity through circulating tumor DNA (ctDNA) and circulating tumor cells (CTCs) analysis [63]. Extracellular vesicles and their molecular cargo (DNA, RNA, microRNA, proteins and lipids) may also provide information on breast cancer heterogeneity and treatment, as they mediate the communication between tumor cells and the microenvironment and carry genetic information that reflects the clonal composition of the tumor [64]. Beyond mutation-based ctDNA assays, the methylation profile of circulating cell-free DNA (cfDNA) is being developed as a tumor-agnostic marker that allows pan-cancer detection and tissue-of-origin inference from a blood sample [65]. More recently, the field has moved from short to long cfDNA molecules and from simple quantification toward fragmentomics, the study of cfDNA fragment sizes, end motifs and nucleosome footprints which carry information on the tissue of origin and on the epigenetic state of the cells releasing the DNA [66]. cfDNA analysis has now entered the era of mechanistic fragmentomics, in which fragmentation patterns are no longer used only as empirical classifier features but are interpreted through the biological processes that generate them, in particular the activity of specific nucleases (such as DNASE1L3 and DFFB) and the chromatin organization of the cell of origin [67]. Because these mechanisms are themselves altered by tumor biology and by treatment, mechanistic fragmentomics could in principle be used in the neoadjuvant setting to monitor the clearance of tumor-derived DNA during therapy, to infer the tissue and cellular origin of residual signals and to complement the histopathological assessment of residual disease at surgery, although such applications remain to be validated in breast cancer.

The evolution of oncological treatment from cytotoxic chemotherapy toward targeted agents and immunotherapy has progressively reshaped the neoadjuvant strategies available for breast cancer, with anti-HER2 agents and, more recently, immune checkpoint inhibitors being integrated into subtype-specific regimens [68].

From a translational perspective, the histopathological variables analyzed here are inexpensive and available in routine practice, but their predictive value is likely to be enhanced when they are integrated with emerging biomarkers. Multi-omics approaches combining genomic, transcriptomic, proteomic and epigenetic profiling, together with liquid biopsy-based markers such as circulating tumor DNA, are being developed to refine the prediction of response to neoadjuvant therapy, to monitor residual disease and to guide escalation or de-escalation of treatment in precision oncology [61]. Digital pathology and machine learning-based quantification of sTILs, cellularity and tumor-bed features may, in parallel, reduce the observer dependence of the variables used in the present study. Future prospective studies should therefore combine standardized RCB and sTILs assessment with treatment-level data, molecular profiling and long-term survival follow-up.

This study has several limitations. First, it was retrospective and single-center. Second, individual-level treatment data were not available: patients received chemotherapy, HER2-targeted therapy, endocrine therapy or combinations thereof according to NCCN recommendations, but the specific regimens, number of cycles and completion of treatment could not be retrieved from the pathology-based database, so that analyses stratified by, or adjusted for, treatment were not feasible. Because treatment allocation depends on subtype, the observed differences in pathological response between subtypes reflect the combined effect of tumor biology and of the therapy received, and the independent contribution of each cannot be quantified from our data; in particular, the comparison of pCR rates between tumors treated with neoadjuvant endocrine therapy and tumors treated with chemotherapy has very limited interpretability, because endocrine therapy produces pCR only exceptionally and its benefit is better captured by other endpoints (such as Ki-67 suppression or the preoperative endocrine prognostic index) that were not available here. Third, pretreatment clinical (cTNM) stage was not available in the pathology-based database and could not be included in the models, and post-treatment pathological stage was not used as a covariate because it is itself a measure of response; residual confounding by stage therefore cannot be excluded. Fourth, follow-up data were limited to vital status at the last database update, without uniformly documented dates of surgery and of last contact and without information on recurrence; consequently, disease-free and overall survival could not be estimated; the vital-status analyses describe crude mortality at a variable follow-up time and are exposed to ascertainment and immortal-time bias. Fifth, post-treatment molecular subtype reassessment was available only in a subset of patients, and paired specimens were not restained in a single batch, so that part of the observed discordance may reflect pre-analytical and analytical variation rather than biological conversion. Sixth, sTILs, RCB and receptor status were assessed in routine diagnostic practice by the reporting pathologists; no formal interobserver reproducibility study (intraclass correlation coefficient or kappa statistics) was performed, and this is a recognized source of variability for these variables. Finally, quantitative histopathological variables such as tumor cellularity, stromal TILs and tumor-bed selection for RCB calculation are partly observer-dependent and may be affected by sampling limitations, especially in heterogeneous or multifocal tumors, and the higher detection of in situ carcinoma and lymphovascular invasion in resection specimens than in biopsies is at least partly a sampling effect. A prospective, multicenter cohort with treatment-level data, clinical stage, centralized blinded pathology review and structured survival follow-up would be required to validate the associations reported here and to build a robust multivariable predictive model.

## 5. Conclusions

In this retrospective single-center cohort, favorable pathological response after neoadjuvant therapy was uncommon, and most patients had moderate or extensive residual disease according to the Residual Cancer Burden system. Pathological response differed according to immunohistochemistry-based surrogate molecular subtype, with RCB-0/I and pCR observed mainly in HER2-positive and triple-negative tumors, whereas Luminal A tumors showed limited pathological response. Baseline stromal TILs and surrogate subtype were associated with response independently of age and grade, and crude mortality at the last follow-up increased with residual cancer burden, with no deaths among patients achieving RCB-0 or pCR.

These findings support the systematic reporting of selected biopsy features, including stromal TILs, tumor cellularity, necrosis, lymphovascular/perineural invasion, and immunohistochemistry-based surrogate subtype, together with standardized RCB assessment in post-neoadjuvant breast cancer specimens. Validation in larger, treatment-stratified cohorts with formal survival analysis is required before these variables can be incorporated into routine predictive models.

## Figures and Tables

**Table 1:** Descriptive statistics of the patient cohort.

Variable	Before NAT	After NAT
Age	64.5 (55–71)	64.5 (55–71)
Histological type		
Ductal NST	114/146 (78.1%)	-
Lobular	12/146 (8.2%)	-
Other/mixed	20/146 (13.7%)	-
Tumor Grade ^†^		
G1	7/134 (5.2%)	13/115 (11.3%)
G2	96/134 (71.6%)	76/115 (66.1%)
G3	31/134 (23.1%)	26/115 (22.6%)
Unclear	12/146 (8.2%)	31/146 (21.2%)
Present in situ component	29/146 (19.9%)	50/146 (34.2%)
Present tumor necrosis	27/146 (18.5%)	17/146 (11.6%)
Present vascular invasion	34/146 (23.3%)	75/146 (51.4%)
Present perineural invasion	6/146 (4.1%)	6/146 (4.1%)
TILs	3 (2–8)	3 (2–9.8)
Tumor cellularity	70 (50–80)	50 (10–80)
Molecular subtype ^†^		
HER2-positive	19/141 (13.5%)	2/58 (3.4%)
Luminal A	27/141 (19.1%)	26/58 (44.8%)
Luminal B	73/141 (51.8%)	19/58 (32.8%)
Mixed/ambiguous	2/141 (1.4%)	1/58 (1.7%)
Triple negative	20/141 (14.2%)	6/58 (10.3%)
Unclassified	5/146 (3.4%)	4/58 (6.9%)
Pathological stage after NAT (ypTNM) ^§^		
Stage 0 (ypT0/is)	n.a.	20/142 (14.1%)
Stage IA	n.a.	25/142 (17.6%)
Stage IIA	n.a.	41/142 (28.9%)
Stage IIB	n.a.	18/142 (12.7%)
Stage IIIA	n.a.	26/142 (18.3%)
Stage IIIB	n.a.	6/142 (4.2%)
Stage IIIC	n.a.	6/142 (4.2%)
Surgical procedure		
Mastectomy	122/146 (83.6%)	-
Sectorectomy	21/146 (14.4%)	-
Type of surgery not specified in the record ^‡^	3/146 (2.1%)	-

Data are presented as median (IQR) or n/N (%); percentages use the number of non-missing observations as the denominator. Pre-treatment variables were assessed on the core-needle/incisional biopsy and post-treatment variables on the resection specimen. ^†^ Post-treatment grade could be assessed only in specimens with sufficient residual invasive carcinoma (n = 115; ‘unclear’ includes the 20 RCB-0 cases and 11 cases with residual tumor insufficient for grading) and post-treatment immunohistochemical subtyping was repeated only in the subset of resection specimens in which it was requested or performed as part of routine diagnostic work-up (n = 58); denominators therefore differ between variables. ^‡^ All patients underwent therapeutic surgery at our institution (a resection specimen was required for inclusion); in three records the type of procedure (mastectomy versus sectorectomy) was not specified in the pathology request form. ^§^ Post-treatment pathological stage (AJCC 8th edition, ypTNM) could be assigned in 142 records; pre-treatment clinical (cTNM) stage was not available in the pathology-based database (n.a.). Abbreviations: NAT, neoadjuvant therapy; NST, no special type; RCB, Residual Cancer Burden; TILs, tumor-infiltrating lymphocytes; ypTNM, post-treatment pathological TNM stage.

**Table 2:** Cross-tabulation of Residual Cancer Burden class and pathological complete response (n = 146).

RCB Class	pCR (ypT0/is ypN0)	Non-pCR	pCR Not Evaluable	Total
RCB-0	18	0	2	20
RCB-I	0	14	0	14
RCB-II	0	64	0	64
RCB-III	0	46	0	46
RCB not evaluable	0	0	2	2
Total	18	124	4	146

Values are numbers of records. pCR, pathological complete response; RCB, Residual Cancer Burden.

**Table 3:** Vital status according to pathological response and baseline features.

Variable	Deceased, n/N (%)	*p*-Value	Firth OR (95% CI)
Pathological response			
RCB-0	0/20 (0.0)		
RCB-I	2/14 (14.3)		
RCB-II	5/64 (7.8)	0.008 (trend: 0.006)	2.52 (1.32–5.69) per class
RCB-III	12/46 (26.1)		
RCB 0/I	2/34 (5.9)		reference
RCB II/III	17/110 (15.5)	0.245	2.43 (0.71–12.69)
Non-pCR	19/124 (15.3)		reference
pCR (ypT0/is ypN0)	0/18 (0.0)	0.131	0.15 (0.00–1.16)
Baseline surrogate subtype			
HER2-positive	0/19 (0.0)		
Triple-negative	5/20 (25.0)	0.118	HER2-positive/TNBC vs. luminal:
Luminal B	8/73 (11.0)		0.95 (0.30–2.62)
Luminal A	6/27 (22.2)		
Baseline features			
Age (years), median (IQR)	63 (56.5–72) vs. 65 (55–71)	0.963	0.99 (0.66–1.54) per 10 years
sTILs low (0–10%)	17/115 (14.8)		
sTILs intermediate (11–59%)	2/17 (11.8)	1.000	1.10 (0.86–1.33) per 5%
sTILs high (≥60%)	0/1 (0.0)		
Grade G1–2	10/103 (9.7)		reference
Grade G3	6/31 (19.4)	0.203	2.27 (0.74–6.54)
Tumor necrosis absent	12/117 (10.3)		reference
Tumor necrosis present	7/27 (25.9)	0.052	3.09 (1.07–8.49)
Lymphovascular invasion absent	13/110 (11.8)		reference
Lymphovascular invasion present	6/34 (17.6)	0.392	1.65 (0.56–4.47)
In situ component absent	15/116 (12.9)		reference
In situ component present	4/29 (13.8)	1.000	1.16 (0.33–3.38)
Ductal NST	15/114 (13.2)		NST vs. other: 0.99 (0.34–3.42)
Lobular	2/12 (16.7)	0.819	
Other/mixed	2/20 (10.0)		

Deaths: 19/146 (13.0%). *p*-values from Fisher’s exact test (Monte Carlo approximation for tables larger than 2 × 2), the Cochran–Armitage test for trend across RCB classes, or the Mann–Whitney U test for age; odds ratios (OR) from univariable Firth penalized logistic regression with profile-likelihood 95% confidence intervals (CI). Abbreviations: NST, no special type; pCR, pathological complete response; RCB, Residual Cancer Burden; sTILs, stromal tumor-infiltrating lymphocytes; TNBC, triple-negative breast cancer.

## Data Availability

Data supporting these findings are available upon request to the corresponding author.

## Abbreviations

AE/EC	doxorubicina + ciclofosfamida/epirubicina + ciclofosfamida
AJCC	American Joint Committee on Cancer
BC	breast cancer
BCT	breast conserving therapy
CAP	College of American Pathologists
CI	confidence interval
CIS	carcinoma in situ
ER	estrogen receptor
HR	hormone receptors
HER2	human epidermal growth factor receptor
IHC	immunohistochemistry
NAT	neoadjuvant therapy
NCCN	National Comprehensive Cancer Network
NET	neoadjuvant endocrine therapy
NST	no special type
OR	odds ratio
pCR	pathological complete response
PR	progesterone receptor
sTILs	stromal tumor-infiltrating lymphocytes
RCB	residual cancer burden
TNBC	triple-negative breast cancer
TCHP	docetaxel-carboplatin-trastuzumab-partuzumab
TILs	tumor infiltrating lymphocytes
TNM	tumor-node-metastasis
WHO	World Health Organization
